# Visualization and data exploration of chromosome conformation capture data using Voronoi diagrams with v3c-viz

**DOI:** 10.1038/s41598-023-49179-x

**Published:** 2023-12-12

**Authors:** Alan M. Race, Alisa Fuchs, Ho-Ryun Chung

**Affiliations:** 1 Philipps University Marburg, Institute for Medical Bioinformatics and Biostatistics, Marburg, 35043 Germany; 2 Max Planck Institute for Molecular Genetics, Epigenomics, Berlin, 14195 Germany; 3 Berlin Institute for Medical Systems Biology, Max Delbrück Center, Berlin, 10115 Germany

**Keywords:** Software, Bioinformatics, Chromatin analysis

## Abstract

Chromosome conformation capture (3C) sequencing approaches, like Hi-C or micro-C, allow for an unbiased view of chromatin interactions. Most analysis methods rely on so-called interaction matrices, which are derived from counting read pairs in bins of fixed size. Here, we propose the Voronoi diagram, as implemented in Voronoi for chromosome conformation capture data visualization (*v3c-viz*) to visualize 3C data. The Voronoi diagram corresponds to an adaptive-binning strategy that adapts to the local densities of points. In this way, visualization of data obtained by moderate sequencing depth pinpoint many, if not most, interesting features such as high frequency contacts. The favorable visualization properties of the Voronoi diagram indicate that the Voronoi diagram as density estimator can be used to identify high frequency contacts at a resolution approaching the typical size of enhancers and promoters. *v3c-viz* is available at https://github.com/imbbLab/v3c-viz.

## Introduction

**Figure 1:**
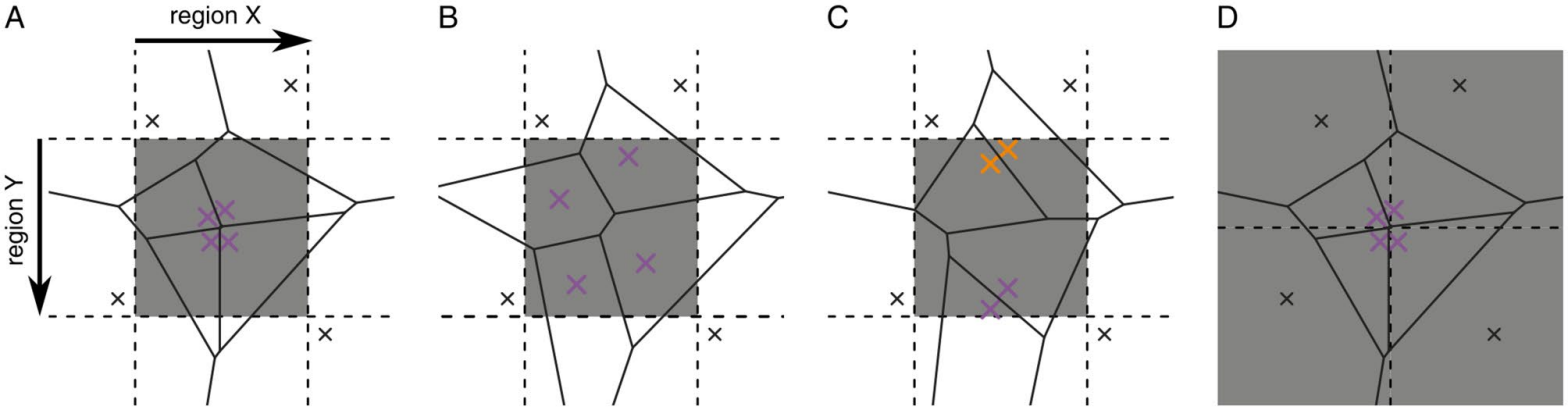
Fixed-bin versus Voronoi diagram. The grey squares correspond to the fixed-bins. The irregular polygons correspond to the polygons of a corresponding Voronoi diagram. In (**A**) there is a centrally located cluster of points. In (**B**) the points in this bin are more evenly distributed. In both cases, we have a count of 4. In (**C**) there are two clusters of points indicated in orange and purple. In (**D**) the cluster of points is located at the corner of four bins.

The development of Hi-C^1^ allowed for probing the three-dimensional conformation of chromatin, genome-wide. The analysis of Hi-C data unraveled chromatin compartments, topologically associating domains (TADs), and contacts between *cis*-regulatory regions^2^. The visualization of Hi-C data was central for the discovery of the aforementioned phenomena. Thus, it is not surprising that a number of tools have been developed for visualizing Hi-C data such as Juicer^3^, GITAR^4^, HiCExplorer^5^ and HiCreekR^6^. These visualizations and also most analysis approaches operate on Hi-C interaction matrices, which are generated by subdividing the plane spanned by two chromosomal regions into equally and fixed sized (square) bins and counting the read pairs that map into region X and region Y (Fig. 1A).

The bin size is a critical hyperparameter that sets the resolution limit of such interaction matrices. All software tools listed above make use of a pre-selected bin size when visualizing or, e.g. in the case of Juicer, binning is performed part of a conversion process to a software-specific data format^7^. Decreasing the bin size leads to a higher resolution. However, decreasing the bin size leads to an unfavorable reduction in the signal-to-noise ratio, such that it is generally perceived that smaller bins require a higher sequencing depth. For the most detailed Hi-C maps, billions of read pairs have been sequenced^8,9^. Since the resolution limit of chromosome conformation capture (3C) is dictated by chromatin fragmentation prior to proximity ligation, restriction enzymes to fragment chromatin have been substituted by more promiscuous endonucleases like DNase (DNase-Hi-C^10^) or MNase (micro-C^11,12^). This alleviates the problem of ‘blind spots’ in regions devoid of restriction enzyme cut sites and increases the fundamental resolution limit to the nucleosomal repeat length of  200 base pairs. However, to realize the so-gained increase in the fundamental resolution limit, billions of fragments have been sequenced^13^.

The fixed-bin approach is not well suited to cope with the spatial heterogeneity present in Hi-C data. This heterogeneity originates from both a distance dependent background contact frequency^1^, as well as from the presence of high frequency contacts, which indicate the presence of stable loops. Here, we propose the Voronoi diagram to visualize 3C data. The Voronoi diagram is an alternative approach to estimate read pair densities to the fixed-bin approach. In the fixed-bin approach we count read pairs in a bin with fixed area, while in the Voronoi diagram we determine the area of an irregular polygon for each read pair, where each point inside the polygon is closer to this read pair than to any other read pair. In both approaches the density is then given by the number of read pairs per area, with the distinction that in the fixed-bin approach the area is constant and the number of read pairs varies while in the Voronoi diagram the number of read pairs is kept constant and the area varies. In this way the Voronoi diagram adapts to the local density of read pairs.

To illustrate this, we prepared two scenarios: four read pairs (marked in purple) are clustered (Fig. 1A) or are more uniformly distributed (Fig. 1B). The fixed-bin approach is not able to distinguish these two scenarios: both central bins have a read pair count of four. By contrast, the Voronoi diagram is able to distinguish between these two scenarios. In Fig. 1A, where the read pairs cluster in the center, the Voronoi polygons are smaller than in Fig. 1B, which can be seen by the ratio of gray-shaded part of the area and white area of each polygon. In the next scenario, there are two read pairs each, which form two local clusters (Fig. 1C; marked orange and purple). In the fixed-bin approach the read pair count is four, i.e. the central bin in this scenario is indistinguishable from the central bins in Fig. 1A and B. The Voronoi diagram constructs polygons, which are smaller than for the uniform case in Fig. 1B pointing to the presence of these two read pair clusters. Finally, high frequency contacts may spread over more than one bin, as illustrated in Fig. 1D. Here, all four bins receive a read pair count of two masking the presence of the four clustered read pairs (marked in purple). The Voronoi diagram constructs polygons for these four points, whose areas are smaller than half of square bin, indicating the presence the cluster.

The fixed-bin approach has obvious limitations that originate from the distribution of read pairs within a bin and from high frequency contacts that span more than one bin. These limitations translate to the visualization properties using interaction matrices based on the fixed-bin approach. To overcome the limitations of the fixed-bin approach we propose Voronoi for chromosome conformation capture data visualization (*v3c-viz*), a user-friendly tool for visualizing 3C data at full resolution (without binning) using Voronoi diagrams. The Voronoi diagram, and its dual the Delaunay triangulation, has application as a non-parametric adaptive density estimator in diverse fields such as social geography^14^, astronomy^15^, and neurobiology^16^. In contrast to machine learning approaches, which aim at predicting interaction matrices with small bin sizes from shallowly sequenced 3C libraries^17–19^, *v3c-viz* is an visualization tool that aims at a visualization of the data as is, with the local density estimate on top.

In the context of 3C-data the Voronoi diagram corresponds to a non-parametric adaptive density estimator, which estimates the contact frequency between two chromosomal regions. In 3C-data contacts are observed by read pairs originating from regions joined by proximity ligation. As alluded to above, every read pair defines a polygon, which consists of points closer to that data point than to any other (Fig. 1). The areas of these polygons are thus inversely correlated to the read pair (contact) density in this region. In fact, the reciprocal polygon area can be viewed as a local density estimate that takes into account all neighboring read pairs. The Voronoi diagram corresponds, therefore, to an adaptive binning approach, where more and smaller bins are used when the local density of read pairs is high and fewer and larger bins are used when the read pairs become sparse. By contrast to the fixed-bin approach, where many bins are zero, any base pair combination of region A to B is inside of a Voronoi polygon. By assigning to all base pair combinations within such a polygon a constant density this means that all possible but mostly unobserved read pairs get assigned a non-zero density.

These characteristics of the Voronoi diagram address the limitations of the fixed-bin approach mentioned above. In the Voronoi diagram it is easy to distinguish between clustered points and evenly distributed points (compare Fig. 1A,B). As it adapts to the local density, it can resolve two distinct clusters (Fig. 1C) and is insensitive to edge-effects (Fig. 1D). Using micro-C data as an example, we demonstrate the favorable visualization properties of the Voronoi diagram.

## Results and discussion

Hi-C methods, such as in situ Hi-C^8^, or the more recent ultra-high resolution method micro-C^13^, allow for detecting chromatin contacts genome-wide. We propose *v3c-viz*, which implements the Voronoi diagram as a non-parametric adaptive density estimator to visualize Hi-C data. *v3c-viz* consists of two parts, a server and a frontend. The server can be run on a local machine for a single-user or on a dedicated server to enable a multi-user system. The server provides a REST API for accessing data, calculating Voronoi diagrams and generating binned representations of the data. The frontend provides a visual representation of the data and enables a user-friendly interface to interact with the data.

Recently, micro-C maps for the embryonic stem cell line H1 and human foreskin fibroblast cell line HFFc6 have been generated^13^. In total 5.89 billion (H1) and 7.23 billion (HFFc6) have been sequenced leading to 3.22 billion (H1 combined) and 5.86 billion (HFFc6 combined) read-pairs in the final data sets. To demonstrate the visualization properties of *v3c-viz* at more shallow sequencing depth, we looked at a subset of the data, namely the first technical replicate for the first biological replicate each, i.e. 496 million (H1B1T1; 15% of the combined data) and 499 million (HFFc6B1T1; 9% of the combined data) read pairs.

**Figure 2:**
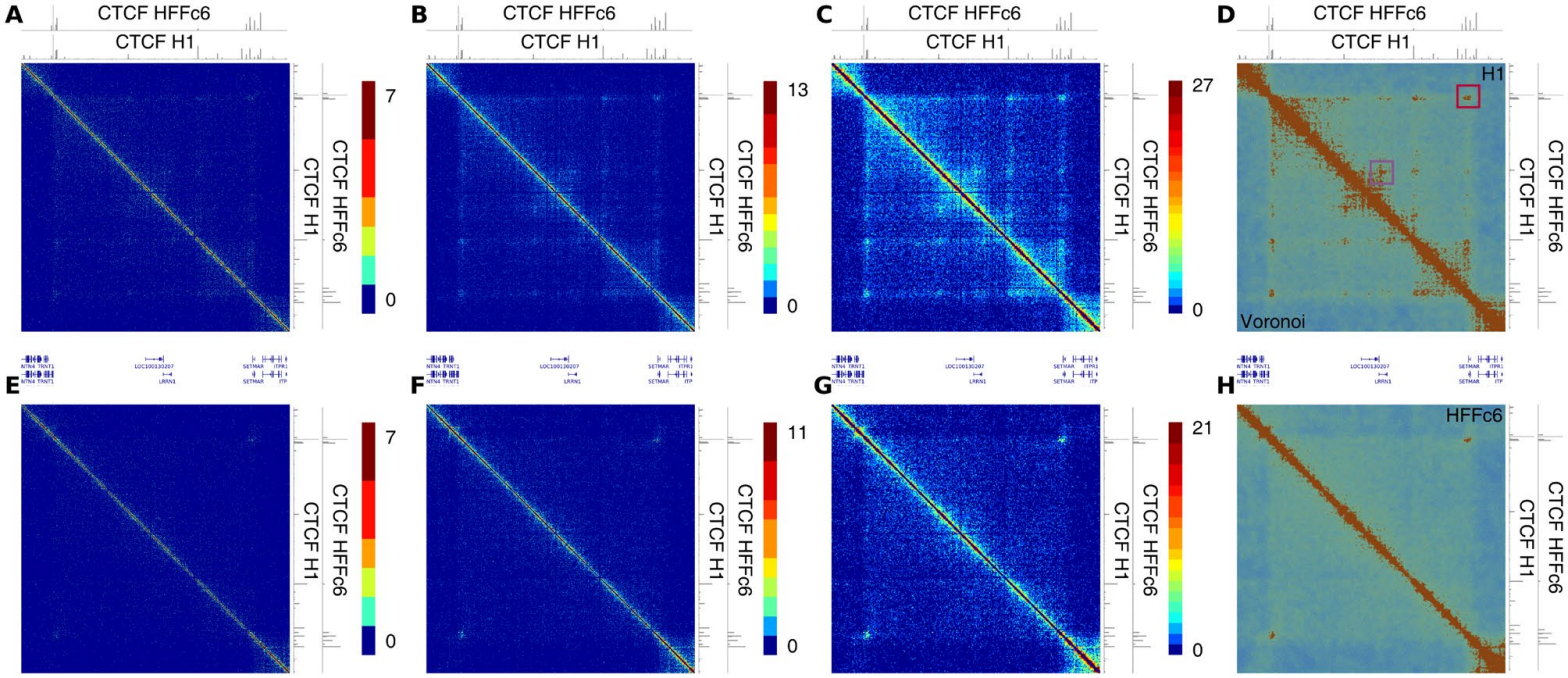
Comparison of fixed-bin versus Voronoi diagram. (**A**) to (**C**) and (**E**) to (**H**) Interaction matrices with a fixed-bin size of 1 (**A**), 2 (**B**) and 5 kb for the H1 B1T1 (**A**) and with a fixed-bin size of 1 (**E**), 2 (**F**) and 5 kb for HFFc6 B1T1 (**G**) data sets; counts on the diagonal and the nearest neighbor bins were removed; the remaining counts were transformed by the square-root function and a saturation point was chosen based on the 99.9% quantile. (**D**) and (**H**) depict the corresponding Vornoi diagrams as exported from *v3c-viz*. The two axes span a region on chromosome 3 from base pairs 3,000,001 to 4,500,000. The tracks on the top and the right correspond to CTCF ChIP-seq signals for the H1 and HFFc6 cell line respectively. The purple square in (**D**) indicates a high frequency contact between the promoter of the LRRN1 gene and a downstream CTCF site.

As an example, we visualized the region on chromosome 3 encompassing base pairs 3,000,000 to 4,500,000, which was also used in Fig. 1 of the original publication of the data^13^. We used a fixed-bin size of 1, 2, and 5 kb to generate heatmaps (Fig. 2A–C,E–G) for the H1B1T1 and HFFc6B1T1 data and show the corresponding Voronoi diagrams as exported from *v3c-viz* (Fig. 2D,H). In the visualization with 1 kb fixed-bins (Fig. 2A,E) the central TAD and focal contacts are hardly visible. Decreasing the resolution to 2 kb fixed-bins somewhat helps to discern the difference of the chromatin contact frequency within the central TAD between H1 and HFFc6. Also, faint dots corresponding to interactions involving CTCF sites in H1 are visible, which, except for the outer-most site, are missing in HFFc6. Upon further increasing the fixed-bin size to 5 kb the TAD structure as well as a number of focal contacts become readily visible.

In the Voronoi diagram for H1 cells, both the TAD as well as a number of dots stand out against the background. These dots connect mostly pairs of CTCF sites bound in H1 cells (Fig. 2D). However, some of them correspond to pairs involving sites not bound by CTCF, e.g. purple square in Fig. 2D, which shows a contact between the promoter of the LRRN1 gene and an upstream CTCF site. In HFFc6 cells, the TAD is also visible but most of the dots within the TAD are not present (Fig. 2H), which can be attributed to the absence of CTCF sites within the TAD in HFFc6 cells. Thus, the Voronoi diagram as implemented in *v3c-viz* allows to visualize both TADs as well as focal contacts comparable to heatmaps using fixed-bins of 5 kb.

**Figure 3:**
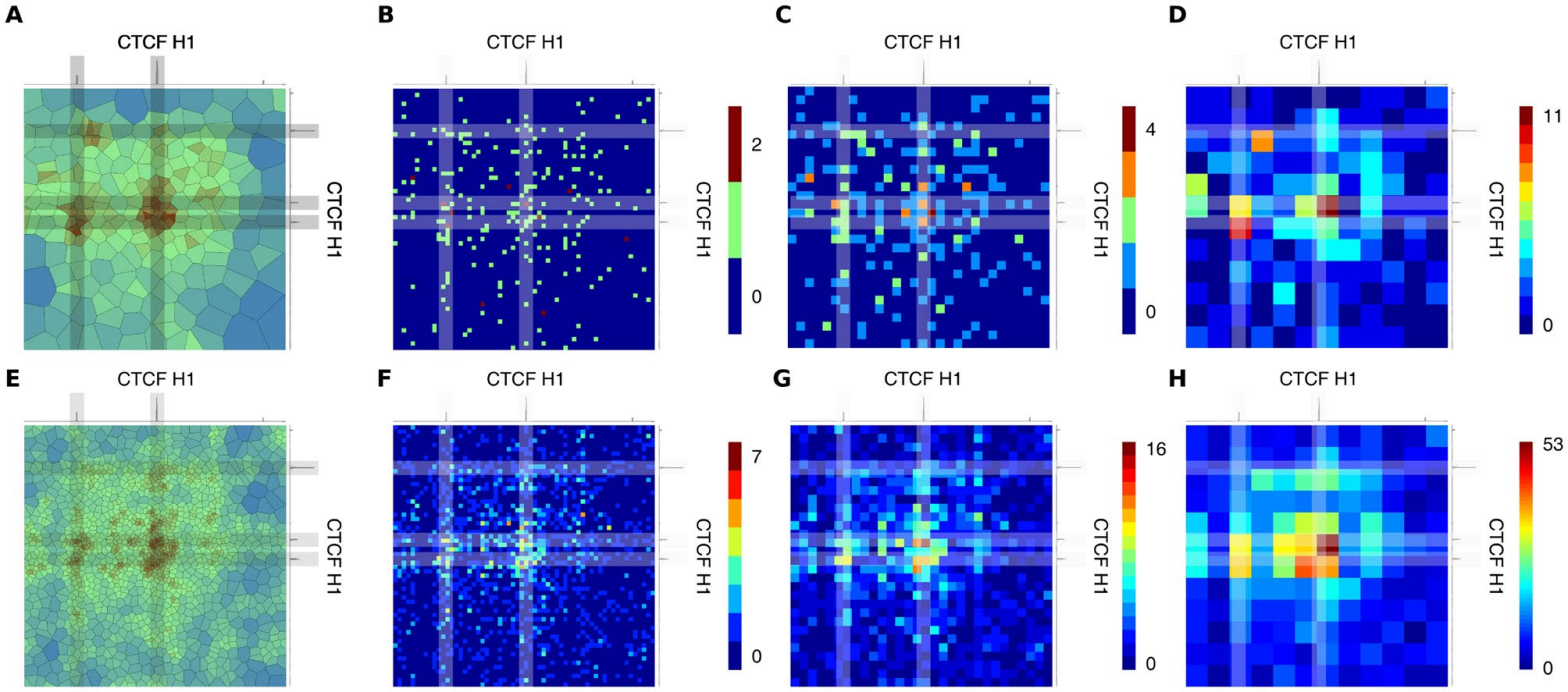
Comparison of fixed-bin versus Voronoi diagram at high magnification. Interaction matrices as well as Voronoi diagrams for the region indicated with a red square in Fig. 2D; the regions on the x-axis correspond to base pairs from 4,260,001 to 4,320,000 and on the y-axis to base pairs from 3,270,001 to 3,330,000. The grey bars indicate regions of CTCF binding. in (**A**–**D**) only the H1 B1T1 data is used. In (**E**–**H**) the H1 combined data is shown.

Using *v3c-viz*, we zoomed into the region indicated by a red square in Fig. 2D. We show the region pair corresponding to the outer-most dot, encompassing the base pairs 3,170,000 to 3,230,000 on the vertical axis and 4,260,000 to 4,320,000 on the horizontal axis (Fig. 3A). This view reveals three to four high density regions. These are well-correlated to CTCF binding sites in H1 cells, indicating that these correspond to CTFC-mediated contacts.

As a comparison we used a fixed-bin size of 1, 2, and 5 kb to generate heatmaps (Fig. 3B–D). In all three resolutions it is very hard to discern the CTCF-mediated contacts readily visible in the Voronoi diagram, which is not really surprising as the maximal read pair number per bin was 2 (1 kb), 4 (2 kb) and 11 (5 kb). At lower fixed-bin sizes of 1 and 2 kb the difference between signal and no signal is very small giving a noisy impression. At the larger 5 kb fixed-bin size the signal increases.

The intricate details of the interactions in this regions are highlighted by the Voronoi diagram for the H1 combined data set (Fig. 3E), where *v3c-viz* is even able to resolve the contacts involving the two closely-spaced CTCF sites on the vertical axis. In the heatmaps at 1, 2, and 5 kb resolution (Fig. 3F–H) the intricate details of the interactions are not readily visible, either due to low signal (1 and 2 kb heatmaps) or due to suboptimal resolution (5 kb heatmap).

Together these examples show that the Voronoi diagram as implemented in *v3c-viz* gives a highly detailed visualization of Hi-C data. The properties of the Voronoi diagram allow to visualize Hi-C data at different scales, megabases to kilobases, giving overviews and highly detailed maps of chromatin interactions. In addition, we would like to stress that we only used one iteration of smoothing and no further pre-processing steps, like normalization or matrix balancing. Thus, the Voronoi diagram shows an unfiltered view on the data and *v3c-viz* allows users to navigate through the data in a meaningful and user-friendly manner.

The Voronoi diagram automatically adapts to the local density of read pairs in 3C data. This property of the Voronoi diagram enables modeling the read pair distribution in a fine-grained and controlled manner alleviating many problems of the fixed-bin approach. The Voronoi diagram uses large bins, when read pairs are scarce and small bins, when there are local clusters of read pairs. Since these local clusters are likely to correspond to focal contacts between CTCF bound region but also between enhancers and promoters, the identification of these focal contacts may help to understand the *cis*-regulatory landscape of a cell.

**Figure 4:**
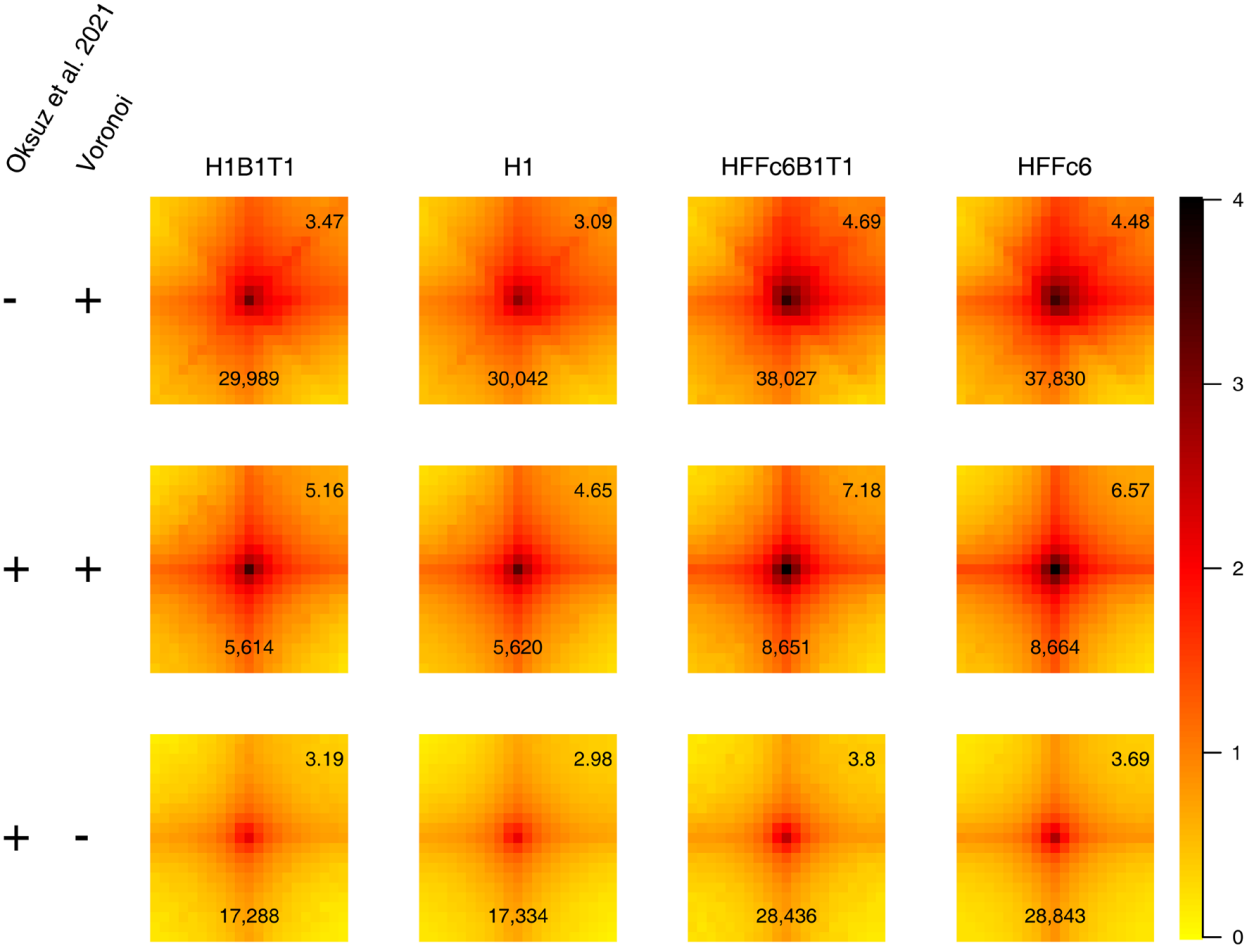
Comparison of high frequency contacts identified in the replicate data H1B1T1 and HFFc6B1T1 using the Voronoi diagram (“Methods”) versus high frequency contacts identified by cooltools using the combined data^20^. Each heatmap represents pileups of the high frequency contacts identified only using the Voronoi diagram (upper row), identified both by cooltools and the Voronoi diagram (middle row) and identified only by cooltools (lower row) for H1B1T1 replicate, H1 combined, HFFc6B1T1 replicate, and HFFc6 combined data. The high frequency contact is at the center of the heatmap, which is extended by ten 5000 bp bins in each direction. Numbers in the top right of each heatmap represent the average signal enrichment at the high frequency contact compared to local background (“Methods”). The number in the bottom of each heatmap shows the number of high frequency contacts with finite signal enrichment. The colors indicate the $\log _2$ average observed over expected signal.

To test whether the Voronoi diagram may help to identify high frequency contacts in data with low sequencing depth, we implemented a rudimentary high frequency contact identification algorithm to identify 5000 bp square bins indicative of high frequency contacts (for details see “Methods”). We applied this algorithm to the replicate data H1B1T1 and HFFc6B1T1 from above and recovered 56,321 (H1B1T1) and 41,826 (HFFc6B1T1) 5000 bp square bins, which we took as candidates for high frequency contacts. We compared these with the high frequency contacts identified by cooltools and reported by Oksuz et al.^20^ for the combined H1 and HFFc6 data (Fig. 4). In total we found 5628 (H1B1T1) and 8670 (HFFc6B1T1) bins overlapping with the cooltools high frequency bins. In addition we found 36,198 (H1) and 47,651 (HFFc6) high frequency bins that were not identified by cooltools. Finally, 17,346 (H1) and 28,862 (HFFc6) high frequency bins identified by cooltools using the combined data were not identified. The common high frequency bins were characterized by the highest signal enrichment (middle row in Fig. 4. The high frequency bins identified only by Oksuz et al. 2021 using the full data show a lower enrichment (bottom row in Fig. 4). The high frequency bins identified only by our rudimentary peak calling algorithm show in all cases a higher average signal enrichment than the ones identified only by Oksuz et al. 2021 indicating that they have at least the “strength” of the loops identified by cooltools using the combined data. We stress that we applied our rudimentary high frequency contact identification algorithm only to 15% (H1) and 9% (HFFc6) of the data.

Taken together, we present a novel approach to visualize 3C data. We make a tool available that allows to use the Voronoi diagram in data mining and to produce publication ready visualizations. Our results for our rudimentary high frequency contact identification algorithm suggest that using the Voronoi diagram as an adaptive density estimator may have advantages over the fixed bin approach, as e.g. implemented in cooltools. Especially, if it is possible to drop the requirements for bins altogether we anticipate that an identification of high frequency contacts at high resolution approaching the typical feature size of promoters and enhancers may be possible—an idea that requires further confirmation in future studies.

## Methods

The software is written in Go (command line tools and server) and TypeScript (interface). Compiled versions for all major operating systems can be found alongside the source code on GitHub (https://github.com/imbbLab/v3c-viz).

Voronoi diagrams are the dual-graph of the Delaunay triangulation, and are therefore calculated directly from the Delaunay triangulation in *v3c-viz*. Delaunay triangulation is computed using a sweep algorithm implementation^21^, which is based on^22–24^.

### Data server

The *v3c-viz* server provides an API for accessing and processing Hi-C data stored in the pairs file format^25^. *v3c-viz* assumes that the data is compressed using the BGZF format (.gz) and the accompanying index file (.px2) enables random access to different loci of the genome without requiring the entire dataset to be loaded in memory when computing Voronoi diagrams. An interact file (see https://genome.ucsc.edu/goldenPath/help/interact.html for details) can also optionally be loaded to visualise contacts or called peaks.

As the API functions over HTTP, it is possible to interact with the server using any programming language, providing a means to integrate the generation of Voronoi diagrams into custom data processing pipelines for the development of further visualization and data analysis algorithms. It is also possible to update the set of contacts or called peaks programmatically so that *v3c-viz* can be used to visualise the results of externally developed methods. Example scripts written in both C++ and the R programming languages showcasing some of these possibilities is supplied alongside the source code. A detailed description of the API can be found on https://github.com/imbbLab/v3c-viz.

### Data visualization

**Figure 5:**
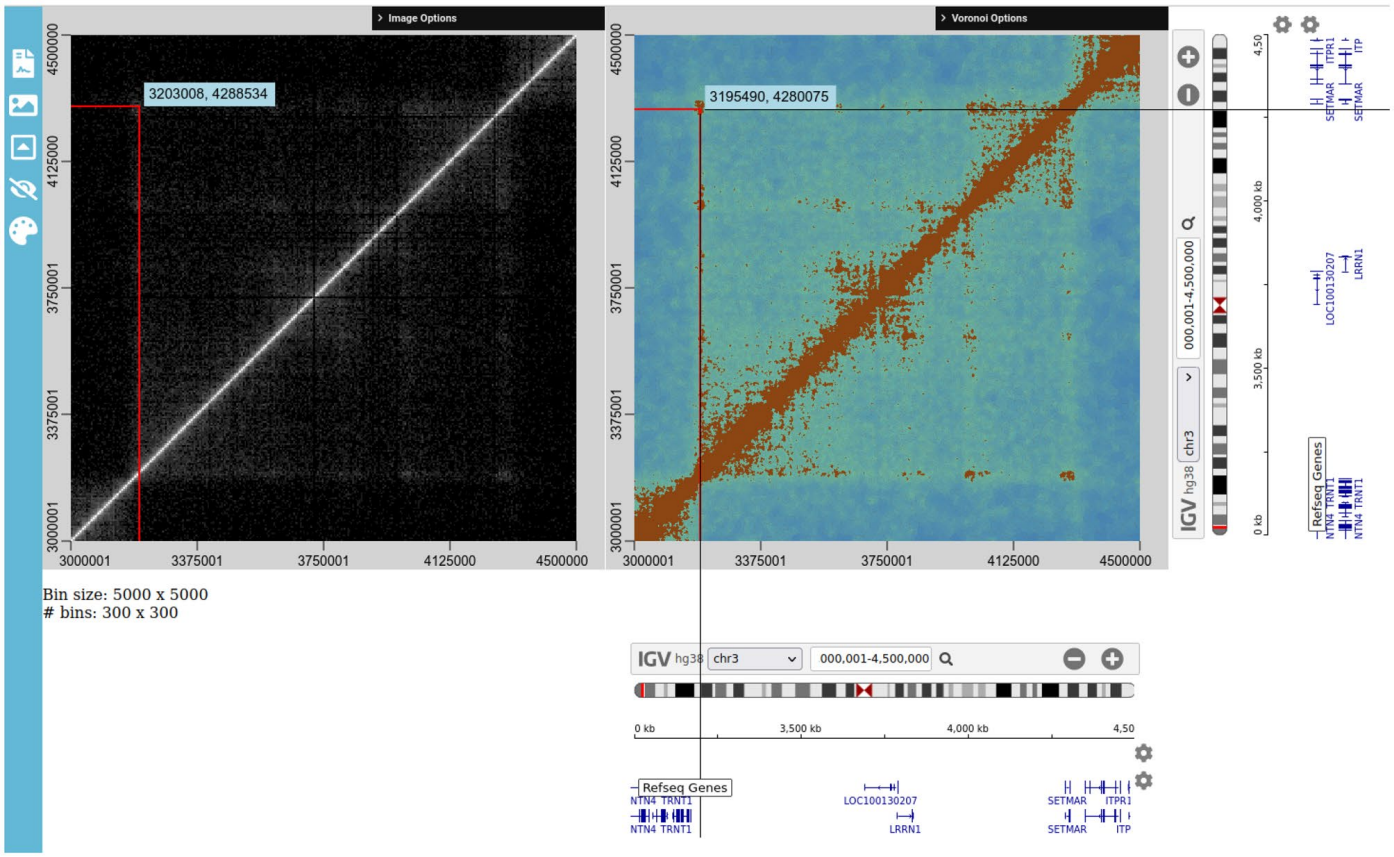
Screenshot of the *v3c-viz* software interface. Two views of the data are shown; (left) binned and displayed as a heat map as is currently done in most software tools and (right) a Voronoi diagram constructed using the data points, with optional smoothing applied, where the Voronoi cells are coloured according to their area. Alongside each axis is an integrated IGV.js browser, allowing visualization of additional genome data.

Once data is loaded, the user is presented with two views of the data; a heatmap representation of the conventional fixed-bin interaction matrix and a Voronoi diagram, as shown in Fig. 5. The heatmap view provides an equivalent representation of the data as is available in existing software tools. The bin size can either be set manually or set to be automatically adjusted based on the size of the current view. As the data is reprocessed on the fly, it is possible to view the data at any resolution without needing to re-convert the data to a software-specific format (as would be the case in Juicer, for example^7^).

Voronoi diagrams created from a set of 2D points consist of one polygon per point, such that the vertices of the polygon are maximally distant from the point and all neighbouring points. Thus, if points are densely packed, the corresponding polygons will have a small area. If points are sparsely distributed the corresponding polygons will be large. This can be seen in Fig. 5, where the colour used to shade each polygon corresponds to its area. This enables the rapid identification of high frequency contacts. The relationship between the contact positions and existing genome annotations can further investigated using the IGV.js browser discussed below.

#### Integrated genomics viewer

It is possible to load in additional information via the integrated IGV.js browser, such as genome annotations (for example in the .bed format), quantitative genomic data (for example in the .wig and .bigWig formats) and genomic variants (in the .vcf format)^26^. A full list of supported track types is kept up-to-date in the IGV.js documentation (https://github.com/igvteam/igv.js/wiki/Tracks-2.0).

Two IGV.js browsers are integrated into *v3c-viz*, one for each of the *x*- and *y*-axes, as visible in Fig. 5. A cursor guide extends from the cursor position and through the IGV.js browsers, enabling the user to determine exactly where in each of the viewed chromosomes the mouse cursor is currently positioned and which parts of the visible tracks in the IGV.js browsers this position corresponds to. All views are kept in sync (including the tracks) as the user selects a region of the chromosome to view. This provides the user with a new means of interactive exploration and interpretation of Hi-C data.

#### Centroidal Voronoi

In many cases, it is easy to overlook possible contacts when inspecting the Voronoi diagram produced from the raw data. An optional feature included in *v3c-viz* is the ability to perform an iterative approximation to the centroidal Voronoi tessellation using Lloyd’s algorithm^27^.

**Algorithm 1:**
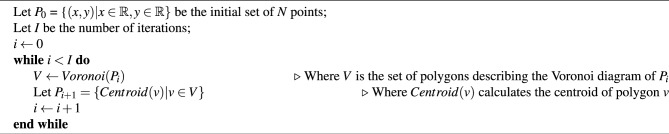
Centroidal Voronoi.

The centroidal Voronoi is described in Algorithm 1. Briefly in words, this works by computing the Voronoi diagram from the original data, as above. Then, for each Voronoi cell the centroid is calculated. The calculated centroids are then used to compute a new Voronoi diagram. This process describes a single iteration, which can be repeated arbitrarily many times or until the newly calculated centroids are co-located with the iteration’s input data points.

**Figure 6:**
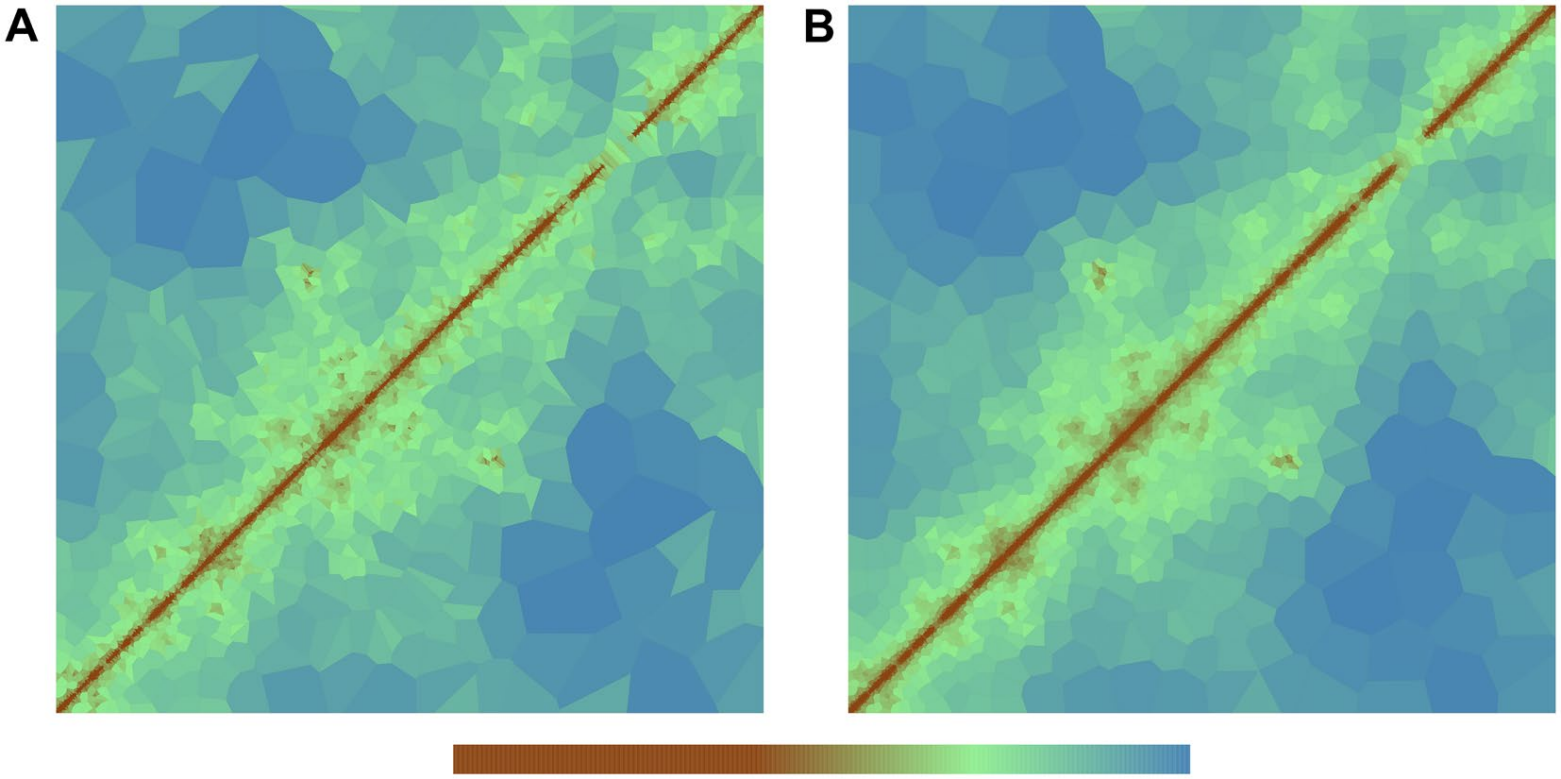
Centroidal Voronoi diagram. Comparison of the Voronoi diagram generated from the raw data (**A**) and with one iteration of centroid Voronoi applied (**B**).

This has the effect of ‘smoothing’ the data, such that resulting Voronoi cells within a neighbourhood have, after each iteration, closer to equal areas. An example of a single iteration can be seen in Fig. 6. Here it is easier, at a glance, to identify potential interactions of interest.

#### Triangle view

**Figure 7:**
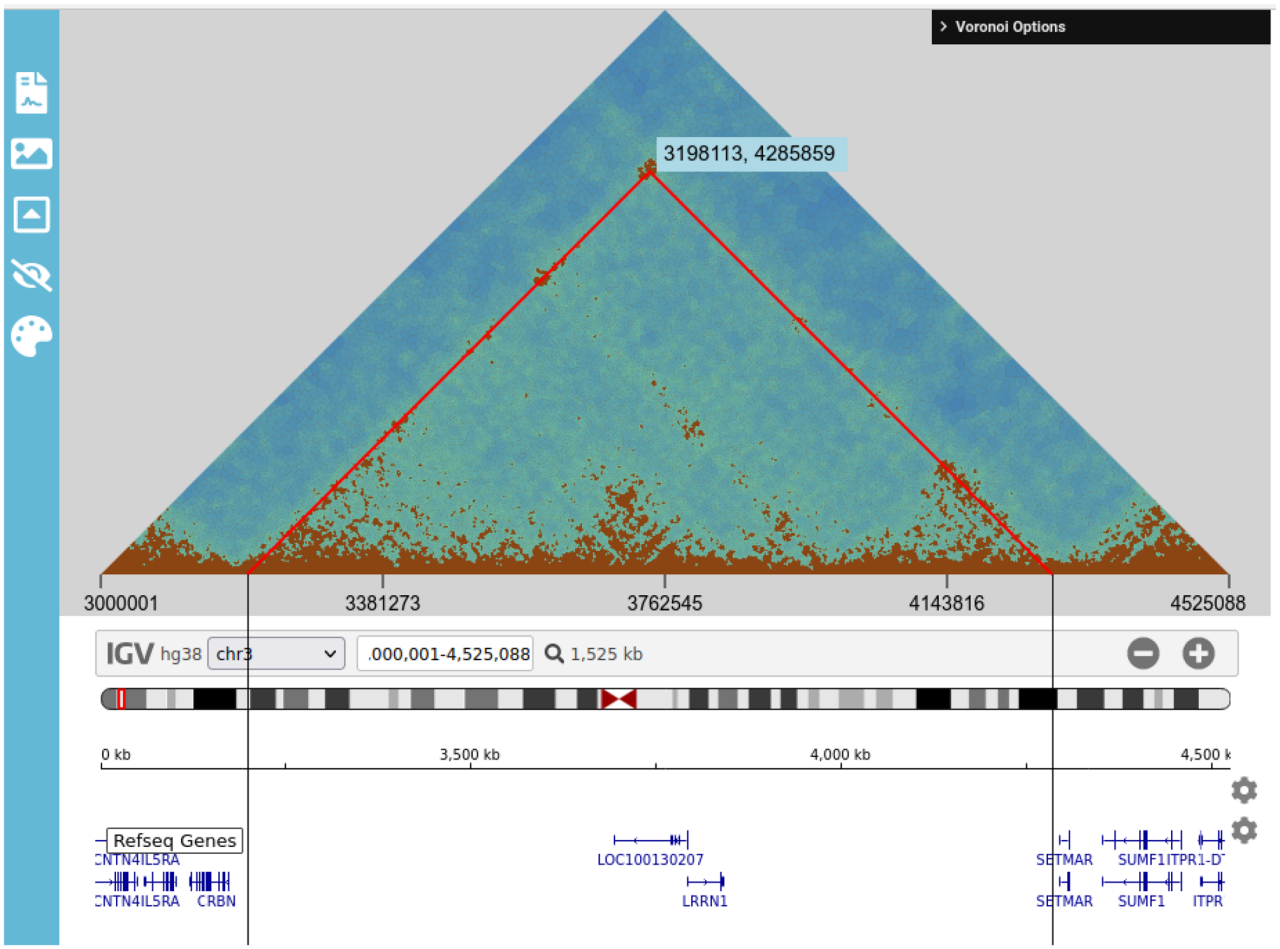
Screenshot of the *v3c-viz* software interface (triangle view).

When viewing intrachromosomal contacts, it is possible to visualise a larger portion of the chromosome when using the triangle view, as shown in Fig. 7. In this view, a single IGV.js browser is included below the Voronoi diagram, otherwise all other functionality works as with the default view.

### Handling large data

The major bottleneck in the visualization is the generation of the Voronoi diagram for large numbers of data points. A number of optimizations are implemented to reduce its impact on the end user. Firstly, a user-specified threshold for the maximum number of points used to generate a Voronoi diagram (default 100,000). If the number of data points exceeds this threshold, then points are binned to the resolution of the output (screen or export file) as Voronoi cells with resolution exceeding this would not be resolvable. If The user then ‘zooms in’, then this binning is recalculated, effectively functioning as an auto-scaling resolution (bin size) for the visualization of Hi-C data.

Secondly, in the case of generating Voronoi diagrams for interchromosomal interactions, is it possible to reduce the number of points in the Voronoi diagram calculation by up to a half, by only calculating the Voronoi diagram for the upper left triangle (see Fig. 5) constrained to this triangle, and then reflecting about the $x = y$ axis to display the full diagram.

Finally, the user can optionally specify a ‘filter distance’ (from the $x = y$ axis), where points that are closer to the $x = y$ axis than the specified distance are ignored from the Voronoi calculation. This can provide orders of magnitude speed-up when investigating long-range interactions, as the majority of data points lie close to the $x = y$ axis and filtering these out reduces the number of data points in the calculation of the Voronoi diagram significantly.

### H1 and HFFc6 micro-C data

Pairs file format files and the corresponding index files for the micro-C experiments for H1 and HFFc6 cell lines^13^ were downloaded from the 4D Nucleome web site (https://www.4dnucleome.org) using the accession numbers 4DNFI8GM4EL9 (H1 B1T1), 4DNFI1O6IL1Q (H1 combined), 4DNFICOEXGPJ (HFFc6 B1T1), 4DNFINYO612N (HFFc6 combined).

### CTCF ChIP-seq data for H1 and HFFc6

BigWig files for CTCF ChIP-seq were downloaded from the Encode Portal (www.encodeproject.org) using the accession numbers ENCFF269OPL (H1 CTCF ChIP-seq) and ENCFF209TQB (HFFc6 CTCF ChIP-seq).

### Identification of high frequency contacts with the Voronoi diagram

We fitted a linear model for the logarithm of the density of each read pair estimated by the Voronoi diagram (without smoothing) as a function of the logarithm of the distance using robust regression as implemented by the R package “robust”. The log-densities predicted by the the distance according to this linear model were taken as an estimate for the expected background density as a function of distance. Read pairs that had a statistical significant higher log density than the background were retained (z-test; one-sided) at an false discovery rate of 10%^28^. We counted these remaining read pairs in 5000 bp square bins and declared arbitrarily those bins with at least 5 read pairs as high frequency bins. Here, we used only the data for the H1B1T1 replicate and the HFFc6B1T1 replicate. Finally, we overlapped the 5000 bp square bins found by this approach with the high frequency bins identified by cooltools and reported by Oksuz et al. 2021^20^. The corresponding high frequency bins were downloaded from the 4D Nucleome web site (https://www.4dnucleome.org) using the accession numbers 4DNFI3RMWQ85 (H1) and 4DNFIIQP46FO (HFFc6).

### Average signal enrichment

For each high frequency contact, we extracted the interaction matrix, where the “expected” signal has been removed using cooltools^29^, at the identified 5,000 bp square bin extended by 50 kb (10 bins) to all directions, resulting in a matrix with 21 rows and columns. The signal strength was than calculated by dividing the average of the pixels (10 to 12 × 10 to 12 with the identified contact in the center) by the average of the “background” pixels in the upper left (1 to 3 × 1 to 3), upper middle (1 to 3 × 10 to 12), upper right (1 to 3 × 19 to 21), middle right (10 to 12 × 19 to 21), and lower right (19 to 21 × 19 to 21). In some cases the average of the “background” pixels was zero, leading to an infinite signal enrichment These were excluded in the calculation for the average signal enrichment.

## Data Availability

The v3c-viz software is available at https://github.com/imbbLab/v3c-viz.
